# IL-17A Facilitates Entry of Autoreactive T-Cells and Granulocytes into the CNS During EAE

**DOI:** 10.1007/s12017-023-08739-0

**Published:** 2023-03-01

**Authors:** Julian Zimmermann, Louisa Nitsch, Marius Krauthausen, Marcus Müller

**Affiliations:** Department of Neurology, University Hospital Bonn, Venusberg Campus 1, 53127 Bonn, Germany

**Keywords:** Interleukin-17, EAE, IL-17A, CNS, Neuroinflammation, Pertussis

## Abstract

Interleukin-17A plays a crucial role in multiple sclerosis and other autoimmune diseases. Although the link between IL-17 and disease activity has been clearly demonstrated, the precise function of this cytokine remains elusive. Here, we investigated the function of astrocyte-targeted IL-17A production in GF/IL-17 transgenic mice during EAE. In particular, IL-17A is important during disease induction. In mice with transgenic IL-17A production, disease occurs earlier and peak disease is more severe, whereas remission is unimpaired. IL-17A synthesis is associated with increased infiltration of granulocytes into the CNS and microglial activation. Moreover, IL-17A synthesis allows induction of MOG-EAE without the additional administration of the co-adjuvant pertussis toxin. Examination of double transgenic GF/IL-17 2D2 mice revealed that, in addition, local IL-17A production facilitates spontaneous infiltration of immune cells into the CNS in mice expressing a MOG-specific T-cell receptor. Overall, we provide evidence for a crucial effect of IL-17A in the induction phase of EAE, facilitating the infiltration of granulocytes and autoreactive T-cells into the CNS.

**Supplementary Information:**

The online version contains supplementary material available at 10.1007/s12017-023-08739-0.

## Introduction

Interleukin-17A (IL-17A) is a key inflammatory cytokine in several autoimmune diseases. Its central role during autoimmune pathogenesis lead to the development of drugs targeting the IL-17A signaling pathway like bimekizumab, secukinumab, or ustekinumab (Mills, 2022). These antibodies are highly effective in treating psoriasis and a clinical trial with secukinumab in multiple sclerosis (MS) provided promising results (Havrdová et al., 2016). During experimental autoimmune encephalomyelitis (EAE), a mouse model of multiple sclerosis, IL-17A plays a pivotal role. Suppression of IL-17A signaling reduces EAE severity and T-cell infiltration (Hofstetter et al., 2005; Hu et al., 2010; Kang et al., 2010, 2013; Komiyama et al., 2006; Veldhoen et al., 2006). On the other hand, however, IL-17A does not mediate direct tissue damage in the CNS, as previously shown (Haak et al., 2009; Zimmermann et al., 2013).

In the context of EAE, IL-17A is primarily secreted from T helper 17 (T_H_17) cells and γδ T-cells (Lalor et al., 2011; Sutton et al., 2006). During MS, IL-17A mRNA is expressed in the cerebrospinal fluid (CSF) of patients (Matusevicius et al., 1999). Furthermore, human T_H_17 lymphocytes promote blood–brain barrier disruption and accumulate in active MS lesions (Kebir et al., 2007). A number of data suggest that especially in the early phase of EAE induction, IL-17A has an important role. Anti-IL-17A treatment reduces EAE severity when given prophylactically or before relapse but not during peak disease (Mardiguian et al., 2013).

In classical EAE models, self-tolerance is broken by immunization against myelin autoantigens like myelin oligodendrocyte glycoprotein (MOG) in the context of a complete Freund’s adjuvant (CFA) (Stromnes & Goverman, 2006). The addition of pertussis toxin (PTX) during immunization is required as a co-adjuvant. The mechanism in which PTX exerts its effect on EAE remains elusive. Besides the hypothesis of increasing the permeability of the blood–brain barrier (Linthicum et al., 1982) several reports point towards the induction of IL-1b and IL-6 by myeloid cells (Dumas et al., 2014; Ronchi et al., 2016). Furthermore, mice with a transgenic T-cell receptor directed against MOG (2D2) also require PTX administration, to develop typical clinical EAE and histological alterations (Bettelli et al., 2003).

To decipher the local impact of IL-17A on neuroinflammation, we generated a transgenic mouse with astrocyte-specific expression of IL-17A under the control of the GFAP promotor (GF/IL-17) (Zimmermann et al., 2013). Our previous studies revealed that CNS-specific IL-17A-synthesis does neither induce any neurological phenotype nor spontaneous demyelination or leukocyte infiltration in the absence of other neuroinflammatory stimuli. However, aging GF/IL-17 mice display microglial activation and local IL-17A synthesis promotes accelerated myelin loss during cuprizone-induced demyelination (Zimmermann et al., 2018). Furthermore, early demyelination was accompanied by an increased ratio of infiltrating granulocytes in GF/ILl17 mice. Here, we induced EAE in these mice in the context of suboptimal immunization protocols and backcrossed the GF/IL-17 mice with 2D2 mice, called GF17-2D2 mice. In this study, we were able to demonstrate that CNS-specific IL-17A synthesis facilitates the induction of CNS autoimmunity and permits spontaneous infiltration of autoreactive T-cells into the CNS.

## Methods

### Animals

The generation of transgenic mice with astrocyte-specific expression of IL-17A under the control of the GFAP promotor, GF/IL-17 mice, has been described in detail previously (Zimmermann et al., 2013). MOG 35–55-specific TCR transgenic 2D2 mice were obtained from Jackson Laboratories (Bar Harbor, ME, USA) and backcrossed to GF/IL-17 mice to obtain hemizygous GF17-2D2 mice. Genotyping of the transgenic offsprings was performed by polymerase chain reaction of tail DNA. All mice were kept under standardized pathogen-free conditions at the animal facility of the University Hospital of Bonn, Germany. Breeding of the animals and animal studies were approved by Governmental Animal Care Commission of North Rhine-Westphalia, Germany.

### Induction and Clinical Assessment of EAE

To investigate the local effect of IL-17A in an EAE model, we immunized 2-months-old WT controls and transgenic animals at an age well before spontaneous microglial activation in the transgenic GF/IL-17 mice occur. On day 0, mice were immunized subcutaneously into the rear flanks with a 1:1 emulsion of 100 μl MOG35–55 or bovine serum albumin (BSA) (3 mg/ml) in 100 μl complete Freund’s adjuvant (CFA) (Sigma-Aldrich, Munich, Germany) supplemented with 4 mg/ml Mycobacterium tuberculosis H37RA (Difco, Detroit, MI) as described before (Quintana et al., 2009). In addition, some mice received an intraperitoneal injection of 500 ng pertussis toxin (Sigma-Aldrich, Munich, Germany) on days 0 and 2.

Animals were examined for 29 days on a daily basis and scored as follows: 0 no clinical symptoms, 1 limp tail, 2 hind limb weakness, 3 hind limb paralysis, 4 hind and forelimb paralysis, and 5 moribund.

### Flow Cytometry

Flow cytometry was performed at indicated timepoints as described previously (Nitsch et al., 2019). Briefly, whole brains were harvested immediately after transcardiac perfusion with ice-cold PBS at the indicated timepoints. One half of the brain was placed in ice-cold Hank’s BSS buffer solution (PAA Laboratories, Coelbe, Germany) and cut into small pieces. Samples were gently homogenized using a Potter–Elvehjem tissue grinder (10 ml; Wheaton), needle (0.6 × 25), and syringe (5 ml) before passing through a 70-μm cell strainer (BD Biosciences). After pelleting, the homogenates were dissolved in 75% Percoll (GE Healthcare). The homogenate mixture was then overlaid with 25% Percoll and PBS. Infiltrating leukocytes and microglia were collected from the 25%/75% interface after an 800 × g centrifugation step for 25 min at 4 °C. For surface marker staining, collected cells were washed in PBS and blocked with CD16/CD32 (Fc block; eBioscience) antibody. The isolated leukocytes were incubated with fluorochrome-conjugated antibody (eBioscience) for detection of CD45, CD3e, CD4, Ly6G, CD11b, Ly6C, B220, and Vβ11-TCR. After washing, surface molecule expression was detected using a BD FACSCanto II (BD Biosciences). Data analysis was performed using FlowJo flow cytometry software (TreeStar).

Before staining and analysis, dissected brain hemispheres were split in 6 different FACS tubes and stained with antibodies or isotype controls. Gating was performed according to supplementary file 1 (sup. Figure 1). Absolute cell counts refer to the entire content of a single FACS tube.

**Fig. 1:**
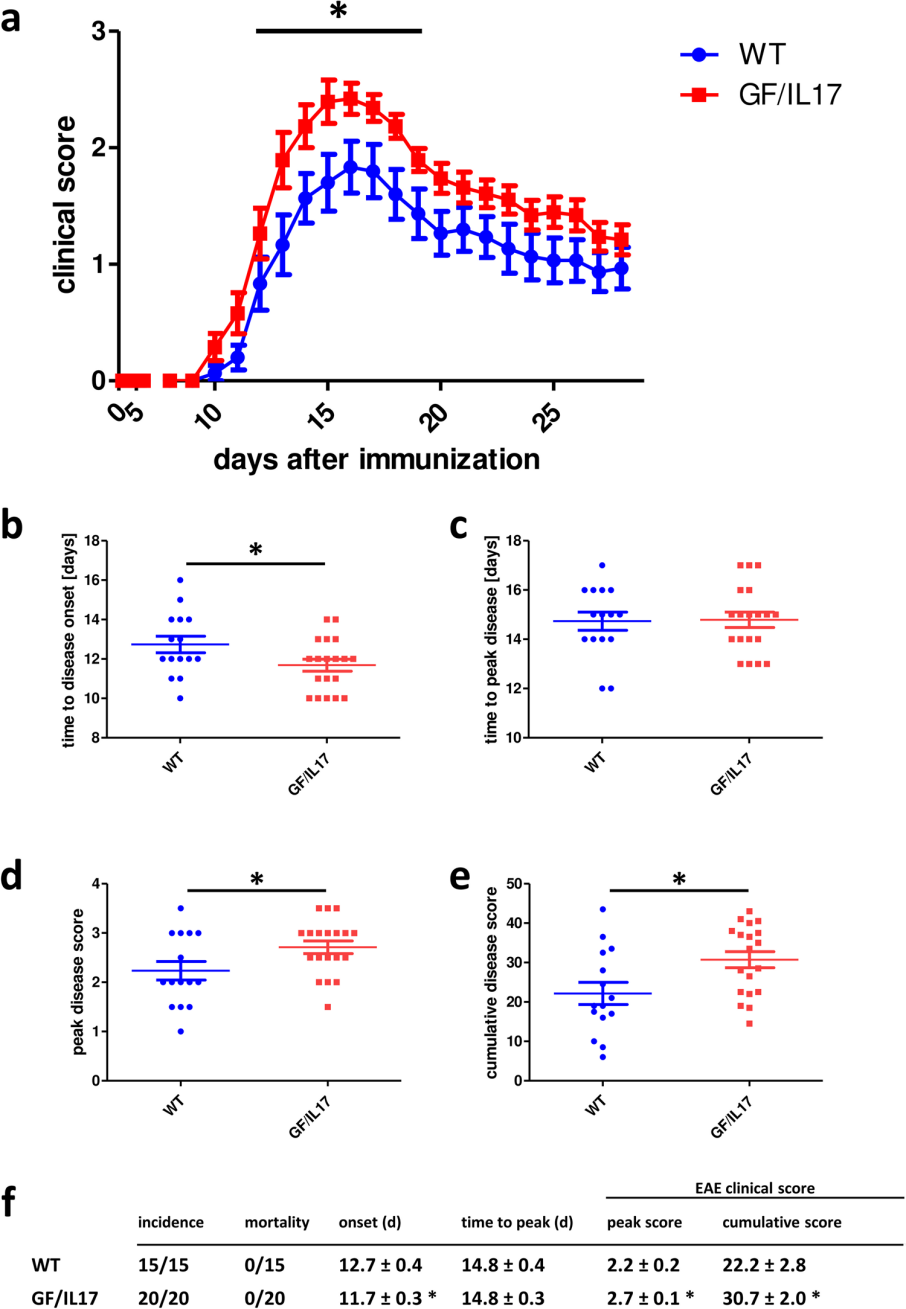
GF/IL-17 mice developed more severe EAE with earlier disease onset and higher peak disease scores. **a** Clinical disease scores after induction of classical MOG-EAE. Dot plots of** b** time to disease onset,** c** time to peak disease,** d** peak disease score, **e** cumulative disease score, and **f** tabular presentation including disease incidence, mortality, average disease onset (EAE score ≥ 1), time to peak disease, and clinical EAE scores in WT and GF/IL-17 mice. Shown are the mean ± SEM, for statistical significance, ∗ , *p <* 0.05 vs WT, daily mean scores, peak score, and cumulative score were analyzed by Mann–Whitney U and time to onset and time to peak score by Student’s *t* test

### IL-17A ELISA

The concentration of plasma IL-17A was determined by ELISA (eBioscience) in EDTA blood of four GF/IL-17 mice and four littermate controls after cardiac puncture and centrifugation according to the manufacturer's protocol. As positive controls served supernatant from primary astrocyte cultures of GF/IL-17 mice in fresh DMEM containing 10% FCS for 12 h as described earlier (Zimmermann et al., 2013).

### Statistical Analysis

All statistical analyses are indicated in the corresponding figures. Data of the clinical assessment were analyzed where appropriate by a Fisher’s exact test, two-sided *t* test or Mann–Whitney U test with a statistical significance defined as *p < 0.05, **p < 0.01, and ***p < 0.001. Statistical analysis of the flow cytometry was performed using a one-way ANOVA followed by an appropriate post hoc test with *P < 0.05, **P < 0.01, ***P < 0.001. All statistical analyses were performed using GraphPad Prism 5.0 (GraphPad Software).

## Results

### CNS-Restricted Expression of IL-17A Leads to an Early Onset and Severe Peak Disease of EAE

To investigate if CNS-restricted expression of IL-17A alters the clinical course of EAE, GF/IL-17 mice and littermate controls were immunized with MOG35-55 at an age of 2 months using a common immunization protocol together with PTX as a co-adjuvant. Nonimmunized (Ni)-WT and Ni-GF/IL-17 mice were clinically indistinguishable at that age.

After immunization, the GF/IL-17 and WT mice developed a typical EAE course with an earlier disease onset in GF/IL-17 mice (day 11.7 ± 0.3) compared with controls (day 12.7 ± 0.4, *p <* 0.05) (Fig. 1). Both GF/IL-17 mice and WT reached peak disease scores at the same timepoint but maximum score was higher in GF/IL-17 mice (2.7 ± 0.1) compared with WT (2.2 ± 0.2, *p <* 0.05).

### CNS-Restricted expression of IL-17A is Not Secreted Into Plasma

**Fig. 2:**
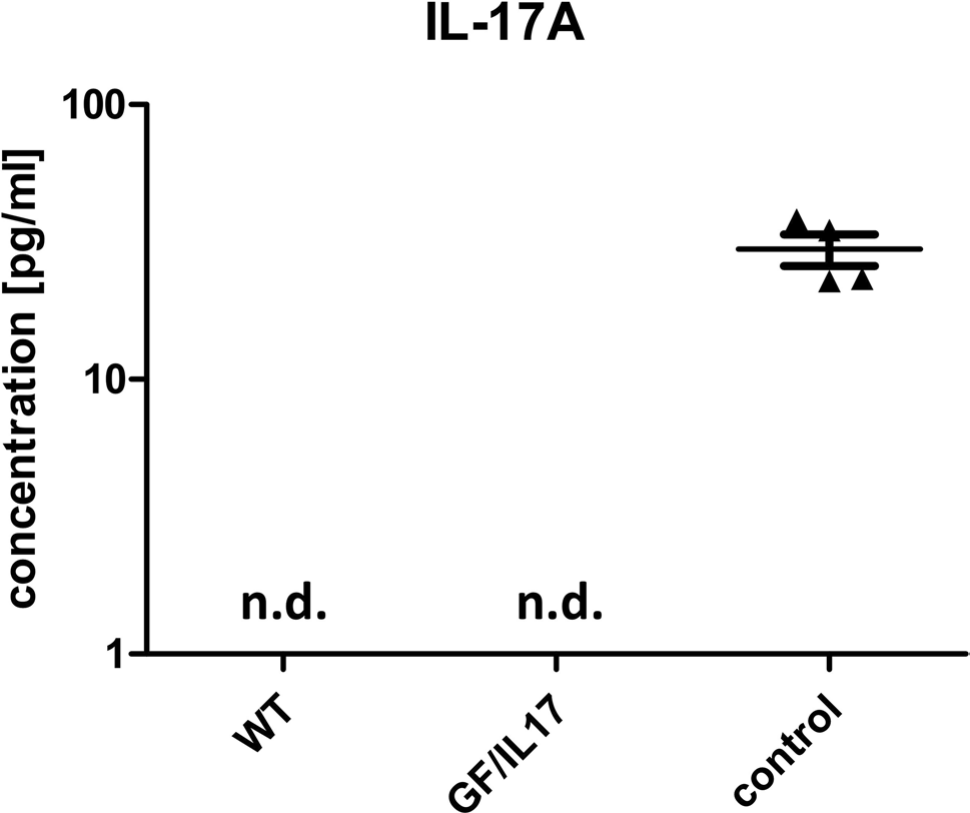
IL-17A protein is not secreted from the brain into the plasma of GF/IL-17 mice. IL-17A was not detectable in the plasma of GF/IL-17 mice (*n =* 4) and controls (*n =* 4) before induction of EAE. As positive control served supernatant from primary astrocyte cultures generated from GF/IL-17 mice. Shown are the mean ± SEM. n.d.: not detectable

In plasma of both transgenic mice and littermate controls, IL-17A protein was not detectable before immunization, respectively (Fig. 2). As control served supernatant from primary astrocyte cultures from GF/IL-17 mice.

### CNS-Restricted Expression of IL-17A Facilitates Granulocyte Infiltration and Microglial Activation During Early Stages of EAE

**Fig. 3:**
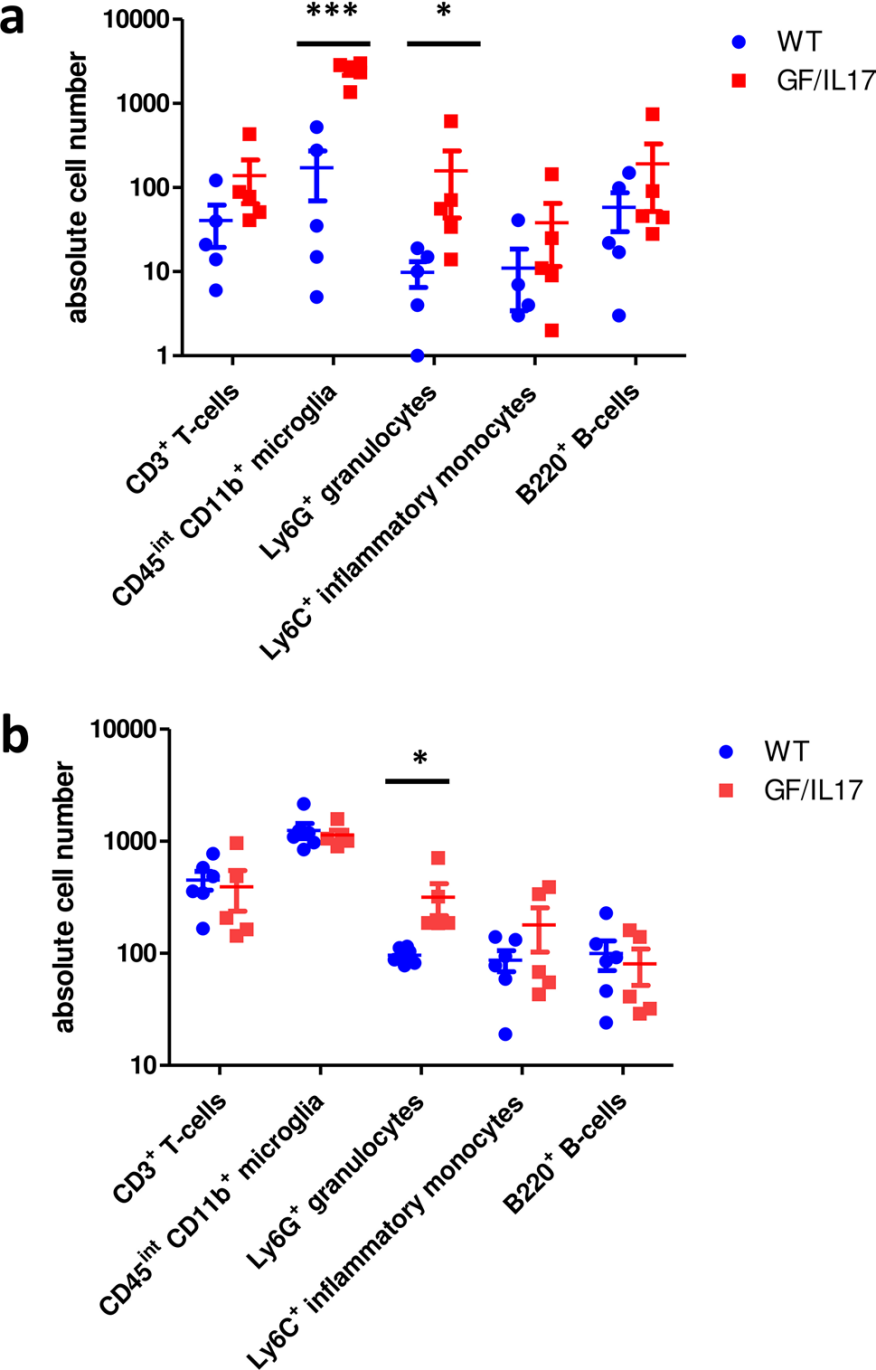
Granulocyte infiltration and increased accumulation of microglia in GF/IL-17 mice at early stages of EAE. Tissue leukocytes were isolated and analyzed by flow cytometry. **a** Cell counts of CD3 + , CD45^int^CD11b^+^, CD45^+^CD11c^+^, Ly6G^+^, and B220^+^ cell populations in WT (*n =* 5) and GF/IL-17 mice (*n =* 5) before disease onset at day 10 after immunization. **b** Cell counts at peak disease, day 15 after immunization (WT: *n =* 6, GF/IL-17: *n =* 5). Shown are the mean ± SEM, for statistical significance, ∗ , *p <* 0.05 vs WT, ∗  ∗  ∗ , *p <* 0.005 vs WT, one -ay ANOVA and unpaired two tailed t-test

The composition of infiltrating immune cells during EAE was characterized by flow cytometry of dissected brains at different timepoints. Before disease onset, already at day ten massive immune cell infiltration into the brains of GF/IL-17 mice and WT was detectable (Fig. 3a). These cells consisted predominantly of infiltrating T-cells, granulocytes, activated microglia, B-cells, and Ly6C^+^ inflammatory monocytes. Interestingly, numbers of infiltrating granulocytes and microglia were massively increased in GF/IL-17 mice compared to WT. 15 days after immunization at peak disease, these differences could only be detected for granulocytes in FACS analysis (Fig. 3b).

### CNS-Restricted Expression of IL-17A Allows EAE Induction Though Suboptimal Immunization

**Fig. 4:**
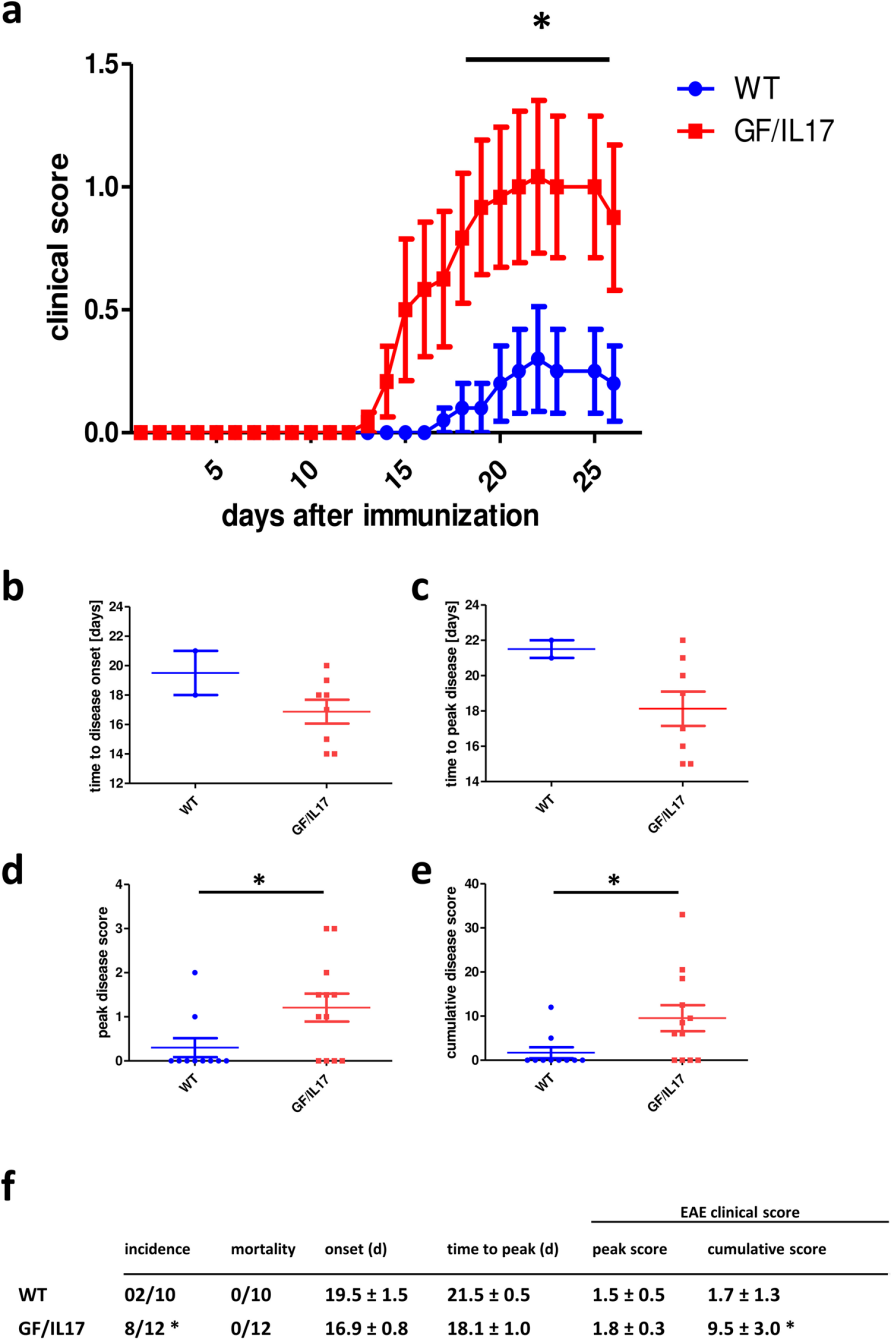
High EAE incidence in GF/IL-17 mice using a suboptimal immunization protocol without PTX **a** Clinical disease scores after induction of suboptimal MOG-EAE without the co-adjuvant PTX. Dot plots of** b** time to disease onset in clinical affected animals only,** c** time to peak disease in clinical affected animals only,** d** peak disease score in all immunized mice, and **e** cumulative disease score in all immunized mice. **f** Disease incidence, mortality, average disease onset (EAE score ≥ 1, only in affected mice), time to peak disease (affected mice only), peak disease score (affected mice only), and mean cumulative EAE scores (all mice) in WT and GF/IL-17. Shown are the mean ± SEM, for statistical significance, ∗ , *p <* 0.05 vs WT, daily mean scores, peak score, and cumulative score were analyzed by Mann–Whitney U and time to onset and time to peak score by Student’s t-test. Incidence was analyzed with Chi-square and Fisher’s exact test

To investigate the impact of local IL-17A synthesis during EAE induction, we performed immunization protocols without PTX in both GF/IL-17 and WT mice, respectively. As expected, WT mice had low incidence rate of clinical symptoms in only 20% of mice with low peak disease scores (Fig. 4). Interestingly, GF/IL-17 mice were susceptible to this suboptimal immunization protocol with an incidence rate of 67% (OR: 8.0, CI: 1.126–56.82, *p <* 0.05). Peak disease scores and disease onset were similar in affected GF/IL-17 and WT, respectively. Cumulative EAE scores including unaffected mice significantly differed between groups. Compared with classical immunization protocol including PTX disease onset was later and disease scores reduced in both groups.

### CNS-Restricted Expression of IL-17A Facilitates Spontaneous Infiltration of MOG-Specific T-Cells

**Fig. 5:**
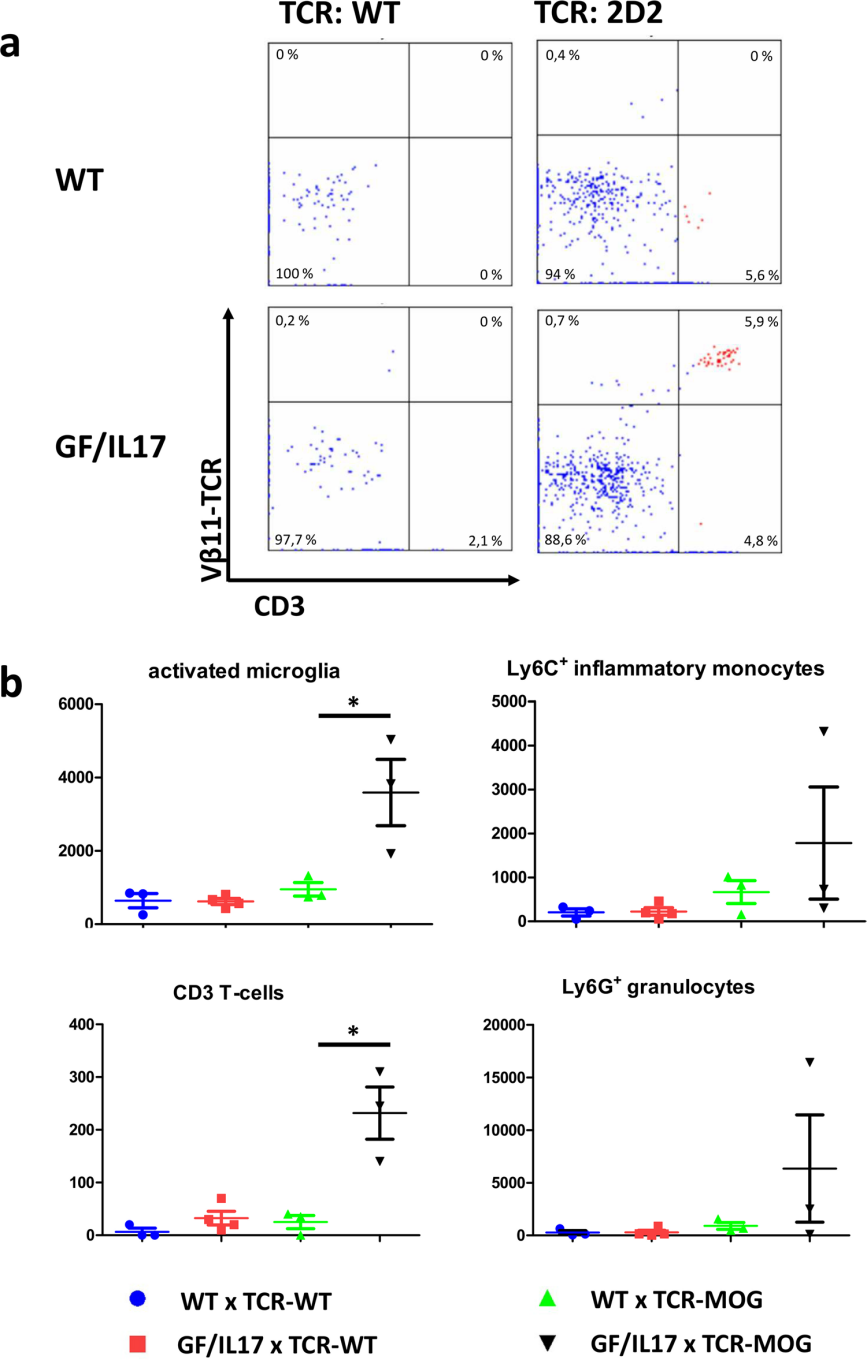
Spontaneous immune cell infiltration into the CNS of GF/IL-17 mice transgenic for a MOG-specific T-cell receptor (GF17-2D2) after 6 months. **a** FACS analysis of immune cells in dissected brains displayed MOG-specific T-cells in GF17-2D2 mice by detection of the transgenic Vβ11 chain on infiltrating CD3^+^ T-cells. **b** Phenotyping of dissected brain immune cells revealed significant T-cell infiltration and microglial activation in GF17-2D2 double-positive mice at the age of 6 months. blue: WT x TCR-WT: *n =* 3, red: GF/IL-17 x TCR-WT: *n =* 4, green: WT x TCR-MOG: *n =* 3, black: GF/IL-17 x TCR-MOG: *n =* 3). Shown are the mean ± SEM, for statistical significance, ∗ , *p <* 0.05 vs WT x TCR-MOG, one-way ANOVA and unpaired two tailed t-test

2D2 mice spontaneously do not exhibit clinical symptoms of EAE or overt immune cell infiltration into the CNS. To investigate the effect of local IL-17A synthesis, we cross-bread 2D2 mice with hemizygous GF/IL-17 mice (GF17-2D2). Neither GF17-2D2 mice, 2D2 mice, nor GF/IL-17 mice developed any clinical symptom of EAE beyond a limp tail within 6 months. Surprisingly, we were able to detect spontaneously infiltrating Vβ11-TCR-positive MOG-specific T-cells only in the brains of GF17-2D2 mice (Fig. 5a). These infiltrations were accompanied by a significant increase in microglia (Fig. 5b). Although some GF17-2D2 mice displayed markedly high numbers of infiltrating granulocytes and activated monocytes, these differences did not reach statistical significance.

## Discussion

IL-17A has been implicated in the pathogenesis of many autoimmune diseases, especially in MS (Mills, 2022). The main sources of IL-17A, T_H_ 17 cells, and γδ T-cells contribute pivotally to MS and EAE (Cua & Tato, 2010; Cua et al., 2003; Langrish et al., 2005; McGinley et al., 2018). Nevertheless, IL-17A alone does not vitally contribute to neuroinflammation as we demonstrated earlier that chronic IL-17A expression in the CNS neither induces demyelination nor leukocyte infiltration (Zimmermann et al., 2013). Ex vivo stimulation of microglia with Th17 cytokines does not influence their activation state (Prajeeth et al., 2014). On the other hand, however, we could show that chronic IL-17A stimulation in the context of other neuroinflammatory diseases, such as a LPS-sepsis model or cuprizone-induced demyelination, enhances neuroinflammatory responses (Zimmermann et al., 2018). In GF/IL-17 mice, toxic demyelination was accelerated and synthesis of myelin proteins was reduced during cuprizone exposure.

The pathogenic role of IL-17A during EAE has been discussed intensively in the literature. However, a study showed that overexpression of IL-17A in CD4 + and CD8 + T-cells does not enhance disease (Haak et al., 2009) others clearly demonstrated the contribution of IL-17A to EAE pathogenesis. Blockade of IL-17A-attenuated EAE disease course as demonstrated in several studies and blood–brain barrier (BBB) integrity is disrupted by IL-17A via reduced expression of tight junction-related genes (Mardiguian et al., 2013; McGinley et al., 2020; Setiadi et al., 2019). Interestingly, T-cells do not need to synthesize IL-17 in the CNS to achieve their encephalitogenic capacity. Gut-targeted production of IL-17 or reconstitution of a wild-type like microbiota in mice with IL-17A/F-deficient T_H_ cells is sufficient to reestablish their susceptibility to EAE again (Regen et al., 2021). Another recent paper links encephalitogenicity of T_H_ 17 cells with the microbial composition of the intestine. Homeostatic SLAMF6 + T_H_ 17 cell populations are converted to pathogenic CXCR6 + T_H_ 17 cells in intestinal tissues, which migrate to the CNS during EAE (Schnell et al., 2021). This is consistent to our findings, as we clearly demonstrated the effects of this cytokine especially at early timepoints of EAE. The first events during EAE are T-cell priming and thereafter disruption of BBB and trafficking of autoreactive T-cells into the CNS (Sospedra & Martin, 2016). Unfortunately, we were not able to differentiate at which of these early points IL-17 synthesis exactly mediates its effect. Nevertheless, we were able to show that chronic IL-17A expression resulted in accelerated EAE onset and higher peak disease scores in classically induced EAE. Remission after peak disease was unchanged in GF/IL-17 and WT mice, as indicated by the parallel clinical course of the two groups. We speculate that in our model, local IL-17 synthesis mediates a direct CNS-specific effect and does not systemically affect T-cell priming. Although priming is IL-17-mediated in the gut, it is also dependent on the gut microbial flora, which is influenced by IL-17 (Regen et al., 2021). A brain microbiome has yet not been demonstrated and we were able to rule out IL-17 secretion from the brain into the plasma (Bedarf et al., 2021). General, well-described effector functions of IL-17A in autoimmune diseases are induction of inflammatory mediators from keratinocytes and fibroblasts (Mills, 2011). Furthermore, IL-17A is believed to disrupt BBB tight junctions and promote central nervous system inflammation through CD4 + lymphocyte recruitment (Kebir et al., 2007). Our results add further evidence to the hypothesis that IL-17A plays a crucial role, especially in the early phase of EAE. Genetically IL-17-receptor-deficient mice are resistant to the induction of MOG-EAE. However, neutralization of IL-17A during the effector phase no longer affects disease progression (McGinley et al., 2020). The same work found high expression levels of the IL-17A receptor IL-17RA on neutrophils and Ly6C^+^-inflammatory monocytes early in EAE. Genetic deficiency of IL-17 in turn leads to ameliorated EAE course and reduced numbers of these cell types in the spleen after EAE induction. Interestingly, we showed in our work that neutrophils and Ly6C^+^-inflammatory monocytes are recruited to the CNS at a particularly early stage by local IL-17A synthesis, and are thus clearly target cell types. Also in Cuprizone-induced demyelination we were able to demonstrate the early recruitment of neutrophils into the CNS by local IL-17A production (Zimmermann et al., 2018).

Local IL-17A synthesis in the CNS was not only able to accelerate the disease development of EAE, but also to facilitate the induction of EAE with a suboptimal immunization protocol without the addition of the co-adjuvant PTX. Though immunostimulatory effects of PTX are widely used in EAE and other models of autoimmune inflammatory diseases for more than half a century, the exact mechanism how this toxin confers its potent adjuvant activity remains elusive (Levine et al., 1966). An early hypothesis was that PTX increased the permeability of blood vessels, particularly the BBB (Kügler et al., 2007; Linthicum et al., 1982; Schellenberg et al., 2012; Yong et al., 1993). Meanwhile PTX was identified to activate pyrin, thereby inducing neutrophil adhesion to cerebral capillaries (Dumas et al., 2014). The same group demonstrated that PTX increases the plasma level of IL-6, which in turn increases the expression of endothelial adhesion molecules and chemokines, leading to the recruitment of leukocytes that patrol the cerebral vasculature by crawling on the luminal endothelial surface (Richard et al., 2011; Roy et al., 2012). Interestingly, a crucial PTX mechanism is the activation of the inflammasome, whereby PTX causes the cleavage of pro-IL-1β into its active form IL-1β. As discussed earlier, IL-17A by itself induces IL-1β synthesis from granulocytes, thereby activating encephalitogenic T-cells (McGinley et al., 2020). Similarly, we demonstrated that in the context of other inflammatory stimuli, IL-1β synthesis is induced in the CNS of GF/IL-17 mice. We speculate that local IL-17A synthesis renders PTX-induced IL-1β synthesis by neutrophils unnecessary and thus facilitates EAE induction.

The same mechanism might be the explanation of the spontaneous recruitment of encephalitogenic T-cells with a MOG-specific T-cell receptor in GF17-2D2 mice. 2D2 mice do not develop spontaneous T-cell infiltration without PTX administration (Bettelli et al., 2003; Waldner et al., 2004). PTX-driven priming of encephalitogenic 2D2 cells for the induction EAE is driven via IL-1β (Ronchi et al., 2016). In the context of chronic CNS-specific IL-17A synthesis, Vβ11-TCR-positive MOG-specific T-cells were spontaneously recruited into the brains of double transgenic mice, accompanied by microglial activation and infiltration of neutrophils and inflammatory monocytes.

In an experimental stoke model, IL-17A drives CXCL-1-mediated recruitment of neutrophils into the CNS (Gelderblom et al., 2012). After cerebral ischemia conventional dendritic cells populate the CNS becoming the major source of IL-23, thereby promoting IL-17 induction in γδ T-cells and neutrophil infiltration (Gelderblom et al., 2018). Though, IL-23 is well known to contribute to the development and differentiation of Th17 cells, CNS-targeted overexpression of this cytokine using the same promoter does not lead to spontaneous granulocyte infiltration into the CNS but to a different phenotype with spontaneous accumulation of T- and B-cells into the brain and the clinical development of cerebellar ataxia (Nitsch et al., 2019). These slightly divergent results suggest a strong context dependence of these cytokines on the disease involved. In the context of EAE, astrocyte-specific expression of IL-23 results in a particular accumulation of B-cells and γδ T-cells, demonstrating that the CNS-specific effects of IL-23 are much more extensive than local IL-17A synthesis (Nitsch et al., 2021). Similar to IL-17A, local IL-23 induces spontaneous infiltration of MOG-specific T-cells, interestingly also displaying high numbers of infiltrating B-cells again (Nitsch et al., 2022). These results again show that IL-23 mediates its effects further upstream compared to IL-17A and that blocking IL-17A could lead to a more targeted MS therapy than blocking IL-23, which failed in a phase II clinical trial to treat patients with relapsing remitting MS (Havrdová et al., 2016; Segal et al., 2008).

Taken together, CNS-targeted expression of IL-17A facilitated induction of T-cell-mediated autoimmunity in the CNS. In the GF/IL-17-mouse, induction of MOG-EAE was accelerated and peak disease aggravated. CNS production of IL-17A was sufficient to induce MOG-EAE without the co-adjuvant PTX and to mediate spontaneous CNS infiltration of MOG-specific transgenic T-cells in a double transgenic mouse model. Our data support the evidence that IL-17A plays an important role in the early phase of EAE and MS, in particular contributing to the infiltration of immune cells into the CNS. Our findings should reinforce efforts in the development of IL-17A-directed therapies to provide more targeted therapeutic options for individualized treatment of MS patients.

### Supplementary Information

Below is the link to the electronic supplementary material.

Supplementary file1 (TIF 4260 KB)

## Data Availability

The data that support the findings of this study are available from the corresponding author upon reasonable request.
