# Supplementary figures and images for: IL-17A Facilitates Entry of Autoreactive T-Cells and Granulocytes into the CNS During EAE
Source: Neuromolecular Med. 2023 Mar 1;25(3):350–9. doi: 10.1007/s12017-023-08739-0 (PMC10514131; doi:10.1007/s12017-023-08739-0)

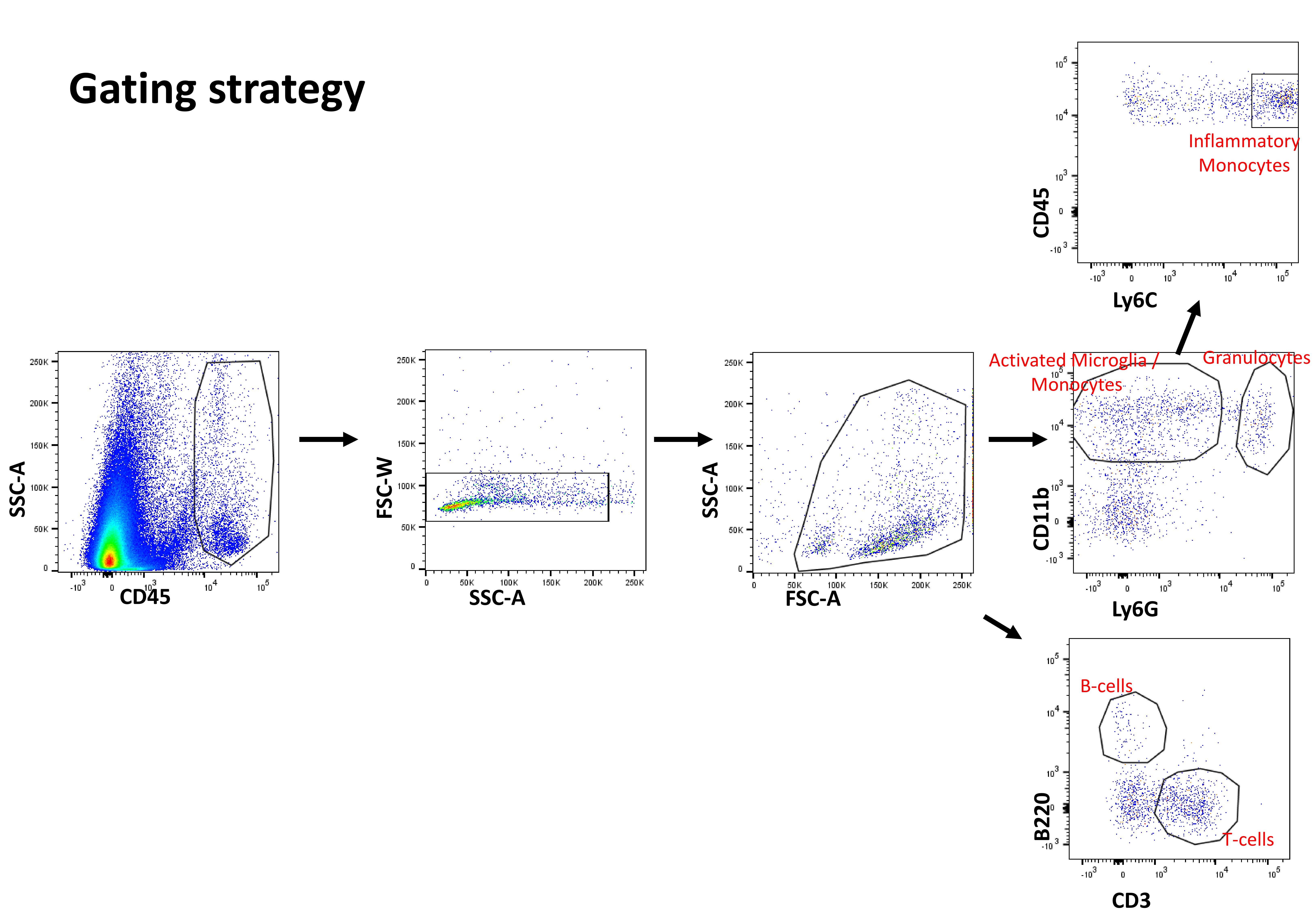

Supplement: Supplementary file 1 — Supplementary file1 (TIF 4260 KB) [file 12017_2023_8739_MOESM1_ESM.tif]
